# Human mesenchymal stem cell basal membrane bending on gratings is dependent on both grating width and curvature

**DOI:** 10.1038/s41598-018-24123-6

**Published:** 2018-04-24

**Authors:** Yukai Zeng, Sum Thai Wong, Soo Kng Teo, Kam W. Leong, Keng-Hwee Chiam, Evelyn K. F. Yim

**Affiliations:** 1 Bioinformatics Institute, A*STAR, Singapore, 138671 Singapore; 2 Department of Biomedical Engineering, National University of Singapore, Singapore, 117583 Singapore; 3 Institute of High Performance Computing, A*STAR, Singapore, 138632 Singapore; 4 Department of Biomedical Engineering, Columbia University, New York, NY 10027 USA; 5 Mechanobiology Institute, National University of Singapore, Singapore, 117411 Singapore; 6 Department of Chemical Engineering, University of Waterloo, Waterloo, ON N2L 3G1 Canada

## Abstract

The topography of the extracellular substrate provides physical cues to elicit specific downstream biophysical and biochemical effects in cells. An example of such a topographical substrate is periodic gratings, where the dimensions of the periodic gratings influence cell morphology and directs cell differentiation. We first develop a novel sample preparation technique using Spurr’s resin to allow for cross-sectional transmission electron microscopy imaging of cells on grating grooves, and observed that the plasma membrane on the basal surface of these cells can deform and bend into grooves between the gratings. We postulate that such membrane bending is an important first step in eliciting downstream effects. Thus, we use a combination of image analysis and mathematical modeling to explain the extent of bending of basal membrane into grooves. We show that the extent to which the basal membrane bends into grooves depends on both groove width and angle of the grating ridge. Our model predicts that the basal membrane will bend into grooves when they are wider than 1.9 µm in width. The existence of such a threshold may provide an explanation for how the width of periodic gratings may bring about cellular downstream effects, such as cell proliferation or differentiation.

## Introduction

Cells sense and adapt to their surrounding environment by responding to biochemical and physical cues. Examples of physical cues include cell-substrate interactions with the topography of the extracellular microenvironment^1,2^, where nanotopography has been shown to regulate of downstream signaling pathways for different cellular functions such as migration, proliferation and differentiation^3–10^. The manipulation of substrate topography is being used in bioengineering applications such as the inducement of bone regeneration^11^, axon guidance^12^ or neuronal differentiation^13,14^. The periodic gratings of polydimethylsiloxane (PDMS) regulate the alignment and elongation of murine neuronal progenitor cells (mNPC)^14^ and human embryonic stem cells in the direction of the gratings. This regulation of cell alignment and elongation occurs in the absence of any chemical cues such as extracellular matrix coatings on the gratings, suggesting that the topographical features of the periodic gratings provide the physical cues required to elicit cell alignment and elongation. Substrate topography is also known to affect the differentiation rate of neuronal progenitor cells into neurons for potential use in cell therapies for neurodegeneration^14–16^. As such, studying the influence of substrate topography on cell response and behavior remains a promising area of research in the field of bioengineering.

It is known that the nanoscale topographical features of the extracellular environment can lead to the differentiation of human mesenchymal stem cells (hMSCs) into different downstream lineages^11,17–22^. For example, during neuronal differentiation of hMSC on gratings, cells elongate and align along the axis of the gratings^23^. As cell shape is one form of physical cues that regulates stem cell differentiation, it can be postulated that the neuronal differentiation of hMSC is regulated by the bending of basal membrane and mediated by the change in cell shape. Detailed investigation of the basal membrane morphology on gratings has been hampered by the limited z-axis resolution of optical microscopy; however, examination of the basal membrane using transmission electron microscope (TEM) was capable to reveal that the membrane bends into the grooves between nano-columns or nano-pillars^24,25^. How topographical features trigger cellular responses, such as differentiation of hMSCs on gratings, is of great interest. Grooves of different sizes may allow the cell basal membrane to bend in between grooves, depending on groove width and curvature. As such, it is important to study the influence of different grating groove features on membrane bending.

In this study, we first develop a novel sample preparation technique using Spurr’s resin as both the substrate and embedding material to create cross-sectional TEM images of hMSCs on gratings. This allows us to observe the regular occurrence of cell basal membranes bending into groove spacing. Further, it is also hypothesized that the extent of this membrane bending is dependent on the width of the gratings and there exist a critical width above which membrane bending will always take place.

Here, the phenomenon of hMSC basal membrane bending into the grating groove was characterized by quantifying the angles at which the membrane dips below the level line at the supporting edge of the ridge. This membrane bending angle was shown to have a dependence on both the groove width and curvature of the supporting edge. Using principles modified from the standard beam bending theory, a membrane bending model was developed to explain this phenomenon of membrane bending. This model can be used to predict if the basal membrane will bend into the grating groove given the grating width and curvature of the grating ridge. Taken together, we develop a novel sample preparation method which allows for the cross-sectional imaging and subsequent experimental quantification of cell basal membrane bending into grating grooves. Together with a membrane bending model, we demonstrate an important role for grating width and curvature of the grating ridge in regulating cell basal membrane bending.

## Methods

### Human mesenchymal stem cell culture

hMSCs were cultured and expanded in MSC growth medium with serum (Lonza, Walkersville, MD). Human MSCs used in experiments were from passage 6–9 and were seeded on gratings and unpatterned Spurr’s resin substrate at a density of 2000 cells/cm^2^. The hMSCs were cultured and maintained under conditions recommended by manufacturer in humidified incubator at 37 °C with 5% CO_2_. The medium was changed every 2–3 days.

### Sample preparation for cell culture

Substrates with gratings of line-width 350 nm, 500 nm, 1 μm, 2 μm and 10 μm were fabricated with Spurr’s resin and polydimethylsiloxane (PDMS). The expected as well as measured line-width, spacing and height of all grating patterns are shown in Table 1. The PDMS mold was fabricated by soft-lithography from gratings on silicon master mold.

**Table 1:** Dimensions of topography of gratings. The expected and measured (mean ± standard deviation) width, pitch and height of the gratings are tabulated.

Grating Dimensions	Gratings Size										
	350 nm		500 nm		1 µm		2 µm		10 µm		n
	Expected	Measured	Expected	Measured	Expected	Measured	Expected	Measured	Expected	Measured	
Line-width (nm)	350	339 ± 7	500	490 ± 20	1,000	981 ± 50	2,000	1,983 ± 65	10,000	9,965 ± 337	7
Spacing (nm)	700	688 ± 22	1,000	966 ± 33	2,000	1,974 ± 52	4,000	3,906 ± 71	20,000	19,422 ± 307	7
height (nm)	350	345 ± 13	350	354 ± 22	350	341 ± 24	350	365 ± 26	350	355 ± 26	4

PDMS base and curing agent (Sylgard 184 Silicone Elastomer Kit, Dow Corning, Midland, MI) were mixed with a 10:1 ratio for 10 min before being degassed in a desiccator for 30 min. The mixture was poured over the silicon master molds, degassed in a desiccator for an additional 30 min and cured at 80 °C for a duration of 12 h. The PDMS substrates were then peeled off from the silicon master molds.

Spurr’s resin (Sigma-Aldrich, St. Louis, MA) was subsequently poured onto PDMS grating mold and cured at 60 °C until hardened. The Spurr’s resin was then peeled off from the PDMS molds by hand. The ability of the PDMS molds to deform during the peeling process allows for the Spurr’s resin to be removed easily^26^. The fidelity of the replication was inspected by scanning electron microscopy (SEM) and atomic force microscopy (AFM). The samples were sputter-coated with a 5 nm coating of chromium and viewed with a LEO Field Emission SEM (LEO 1550, LEO Electronic Microscopy Inc., Thornwood, NY) at 1 kV. Atomic force microscopy surface characterization was performed using contact mode scanning probe microscope (Digital Instrument Dimension 3100, Veeco, Santa Barbara, CA) in the Shared Materials Instrumentation Facility at Duke University. The line-with and spacing of the gratings were examined by multiple measurements on the SEM images (n = 7). The height of were made AFM and TEM (n = 4).

The PDMS samples and Spurr’s resin samples were sterilized under with UV irradiation (UVC germicidal lamps, 254 nm, ~125 µW/cm^2^) in biosafety cabinet for 30 minutes, and coated with collagen I (bovine collagen I, BD Biosciences) at 20 μg/cm^2^ before seeding with hMSC. After 7 days of culture, hMSCs cultured on gratings were washed with pre-warmed serum-free medium, and fixed in 2.5% glutaraldehyde and 4% paraformaldehyde for 45 minutes. The PDMS samples and one set of the Spurr’s resin samples was stained with Oregon Green 488 phallodin (Invitrogen, Carlsbad, CA) and 4′,6-Diamidino-2-phenylindole (DAPI, Invitrogen) for F-actin and nuclei, respectively, to visualize the cell morphology. Cell morphology was observed with epi-fluorescence microscope. An elongation parameter is used to describe the extent to which the equimomental eclipse of the cell body is lengthened^17^, and was calculated by taking the ratio of the long axis over the short axis, minus one. Alignment is defined as the degree in which the long axis of an elongated cell (with an elongation parameter of at least 4) is oriented, with respect to the grating. If the angle between the long axis of a cell and the groove grating was <15°, the cell were considered aligned. For each grating sample, an average of 80 cells was counted.

Gratings samples exhibit a range of substrate bending angles as a consequence of the fabrication process. Spurr’s resin gratings have an elastic modulus in the order of Gigapascals^27,28^.

### Preparation for transmission electron microscopy (TEM)

Another set of hMSC-seeded samples was prepared for TEM. The samples were washed with sodium cacodylate buffer and post-fixed post-fixation in 1% osmium tetraoxide for 1 hour and en bloc stained with 2% aqueous uranyl acetate before dehydrated in a graded ethanol series. Infiltration of Spurr’s resin was initiated with an ethanol/resin mixture in a 1:1 followed by 1:2 ratio. Finally the samples were embedded in resin with the orientation that the sectioning surface was perpendicular to the grating axis. The samples were embedded with 2 changes of resin and cured at 60 °C until hardened.

The embedded samples were trimmed and sectioned to a thickness of 90 nm using the ultratome cutter (Duke University School of Medicine Research Electron Microscopy Service, Durham, NC). The samples were sectioned perpendicular to the grating axis to obtain a cross-sectional view of the gratings and the aligned cells. After section, samples were imaged with a transmission electron microscope (CM-12, Philips/FEI, Hillsboro, OR, USA) at 80 kV. As the 5 mm wide samples were sectioned, a population of cells sectioned at the center and the edge would be observed. Cross-sectional images were taken both at the center and at the edge of cells. The nucleus was used as a reference as the size and height of the hMSC nuclei have been previously characterized^18^. Specifically, the cell height and width of the cells, whether the nucleus was observed, and the width and height of the nucleus were used as an indicator if the section was made at the center or at the edge.

To ensure that the integrity of hMSC and substrate grating samples remain intact after the sectioning process, the integrity of the intracellular structures in hMSCs were observed before accepting the samples for the subsequent TEM measurement (see Supporting Material).

### Basal membrane bending characterization

**Figure 1:**
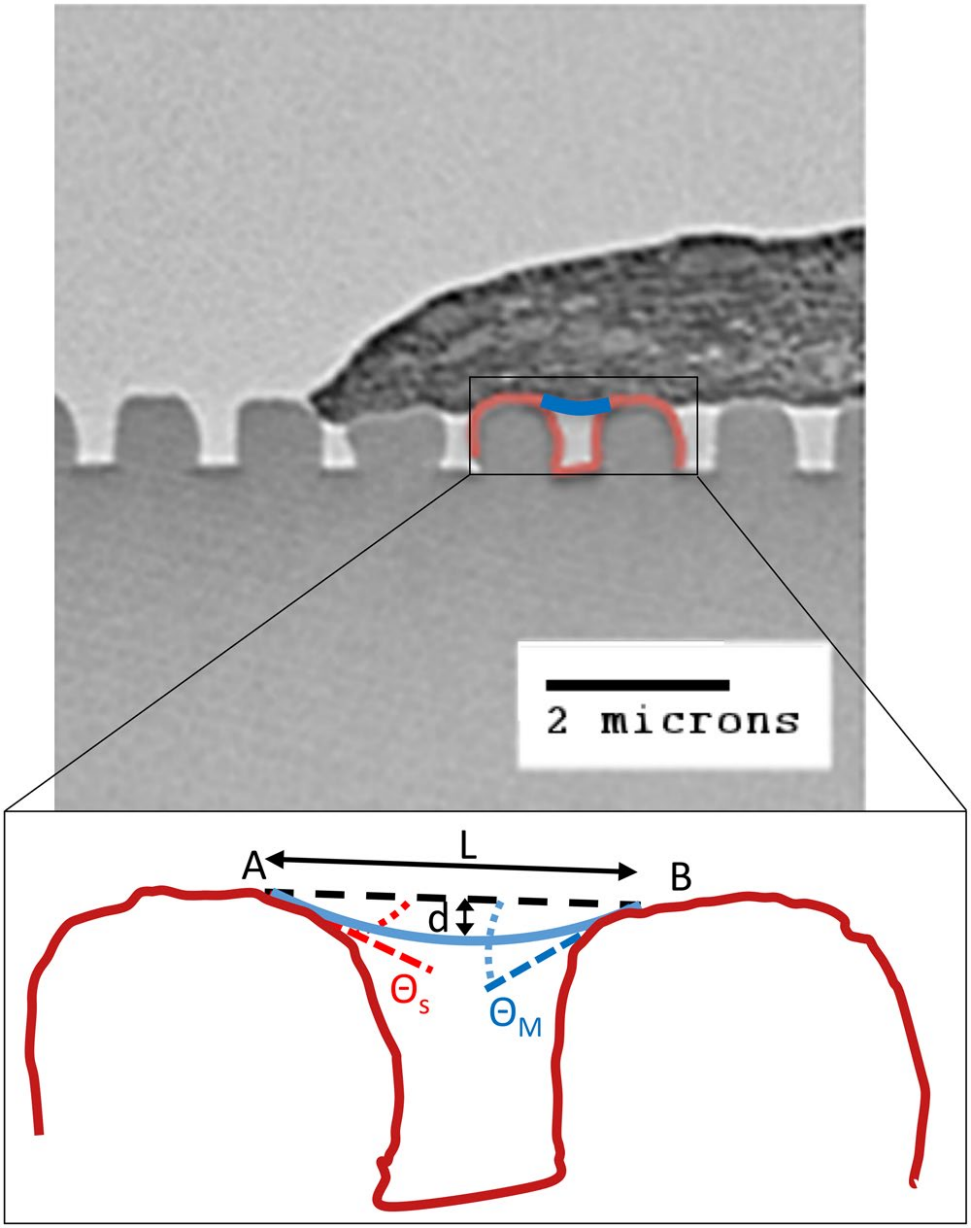
Characterization of basal cell membrane deflection into grating grooves. The following quantities were measured between adjacent ridges: gap width (*L*), maximum deflection (**d**), membrane bending angle (*θ*_*M*_) and substrate bending angle (*θ*_*S*_).

For each TEM image showing the cross-section of hMSC basal membrane bending, we measured the dimensions of the cell membrane deflection within the grating gaps (Fig. 1). A straight line is drawn between the points where the cell membrane comes into contact with the top surface of adjacent grating ridges (Point *A/B*). This zero-deflection line represents the initial position of the membrane before deflection and its length, denoted by *L*, is the grating gap width. The maximum membrane deflection, denoted by *d*, is the distance from this line to the lowest point of the membrane. In the bent state, the angle bounded by the zero-deflection line and the tangent to the membrane at point *A/B* is taken to be the membrane bending angle (*θ*_*M*_). Similarly, the angle bounded by the zero-deflection line and the tangent to the top surface of the grating ridge at point *A/B* is taken as the substrate bending angle (*θ*_*S*_). This measurement is performed for all grating grooves over which the cell membrane spans the full width of the gap. Additional details detailing the calculation of θ_*S*_ and *θ*_*M*_ can be found in the Supporting Material.

## Results

### Characterization of the Spurr’s resin topography and the cell morphology on the gratings

**Figure 2:**
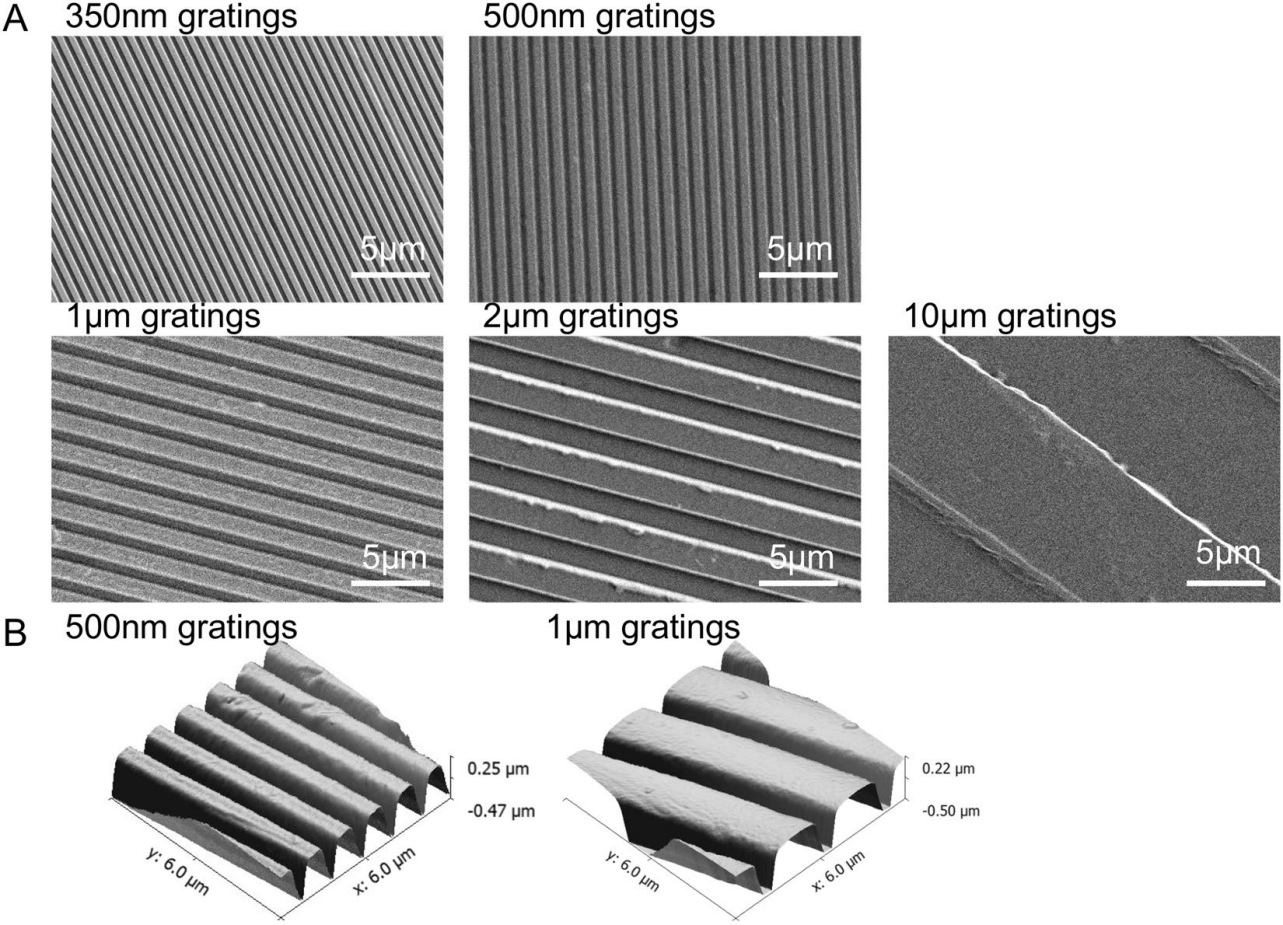
(**A**) Scanning electron micrographs of gratings with 350 nm linewidth and 700 nm pitch (350 nm gratings), 500 nm linewidth and 1 μm pitch (500 nm gratings), 1 μm linewidth and 2 μm pitch (1 μm gratings), 2 μm linewidth and 4 μm pitch (2 μm gratings) and 10 μm linewidth and 20 μm pitch (10 μm gratings) fabricated by casting Spurr’s resin on PDMS mold. (**B**) Atomic force micrograph (AFM) of 500 nm gratings and 1 μm gratings on Spurr’s resin.

**Figure 3:**
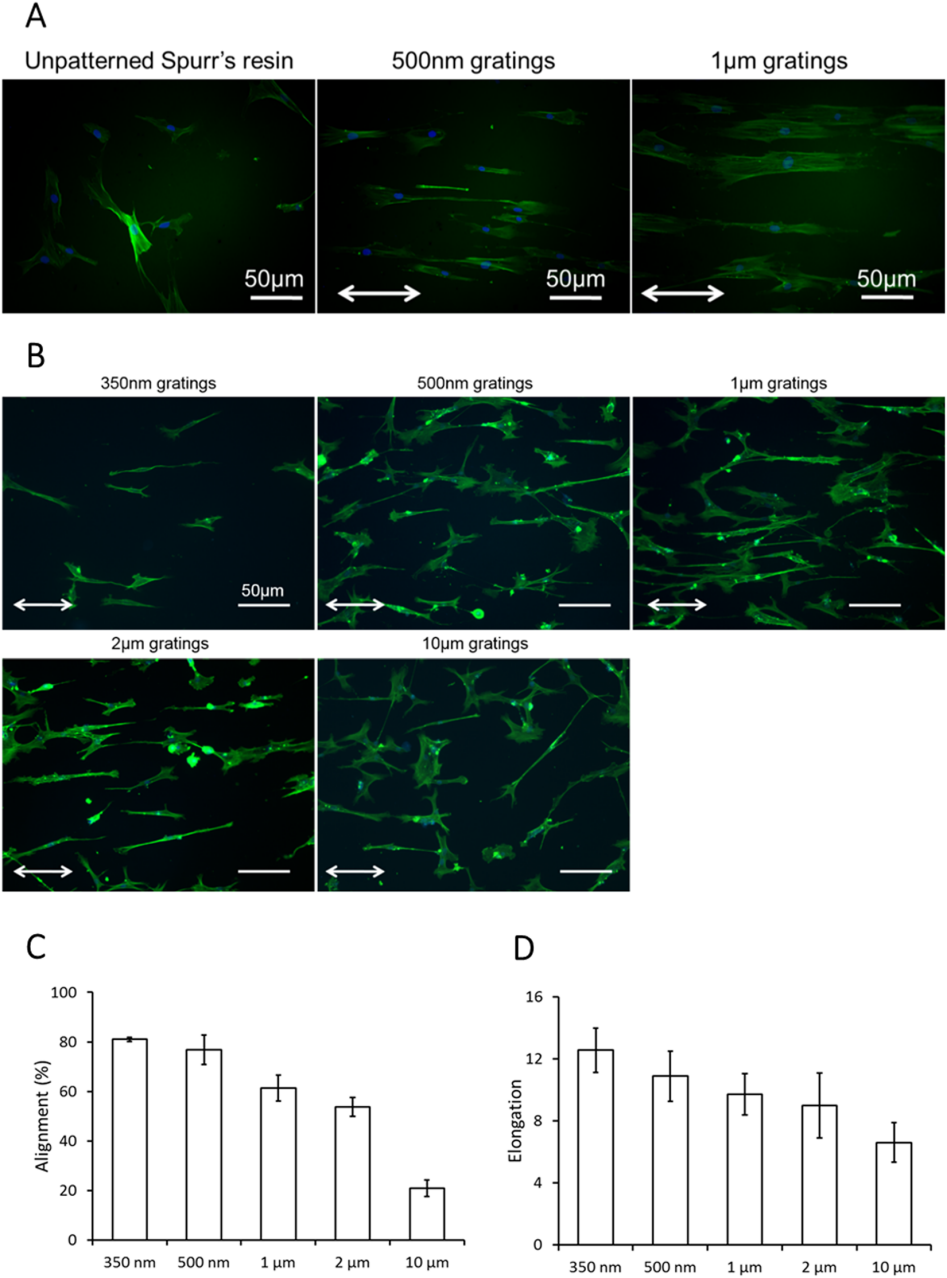
F-actin cytoskeleton visualized by Oregon-green labeled phallodin with DAPI as counter-stain for cell nuclei in hMSC on (**A**) unpatterned Spurr’s resin, Spurr’s resin with 350 nm gratings and 500 nm gratings, (**B**) PDMS with 350 nm, 500 nm gratings, 1 µm, 2 µm and 10 µm gratings. (Bar = 50 μm. Images are taken with fluorescence microscopy. White arrows denote the grating axis direction.) Effect of groove grating width on hMSC morphological properties. Changes in cell body (**C**) alignment and (**D**) elongation of hMSCs cultured on PDMS gratings with different groove widths. (Bars denote standard deviation).

The fidelity and quality of the gratings on Spurr’s resin were characterized by AFM and SEM (Fig. 2). Feature sizes were preserved on Spurr’s resin samples in the replication progress. The en bloc uranyl acetate stained the cells and also the Spurr’s resin substrate, which enable the visualization of the patterned Spurr’s resin substrate in the Spurr’s resin sections (Fig. 1). Alignment and elongation of cells were previously observed on the gratings^15,29^. F-actin cytoskeleton of the hMSCs on the Spurr’s resin and PDMS was visualized by phalloidin (Fig. 3A,B), showing the hMSCs were elongated and aligned along the grating axis of the Spurr’s resin samples and PDMS samples. The extent of elongation, alignment increases with decreasing groove width from 10 µm to 300 nm (Fig. 3C,D).

### Cell membrane of hMSC bends into grating gap

**Figure 4:**
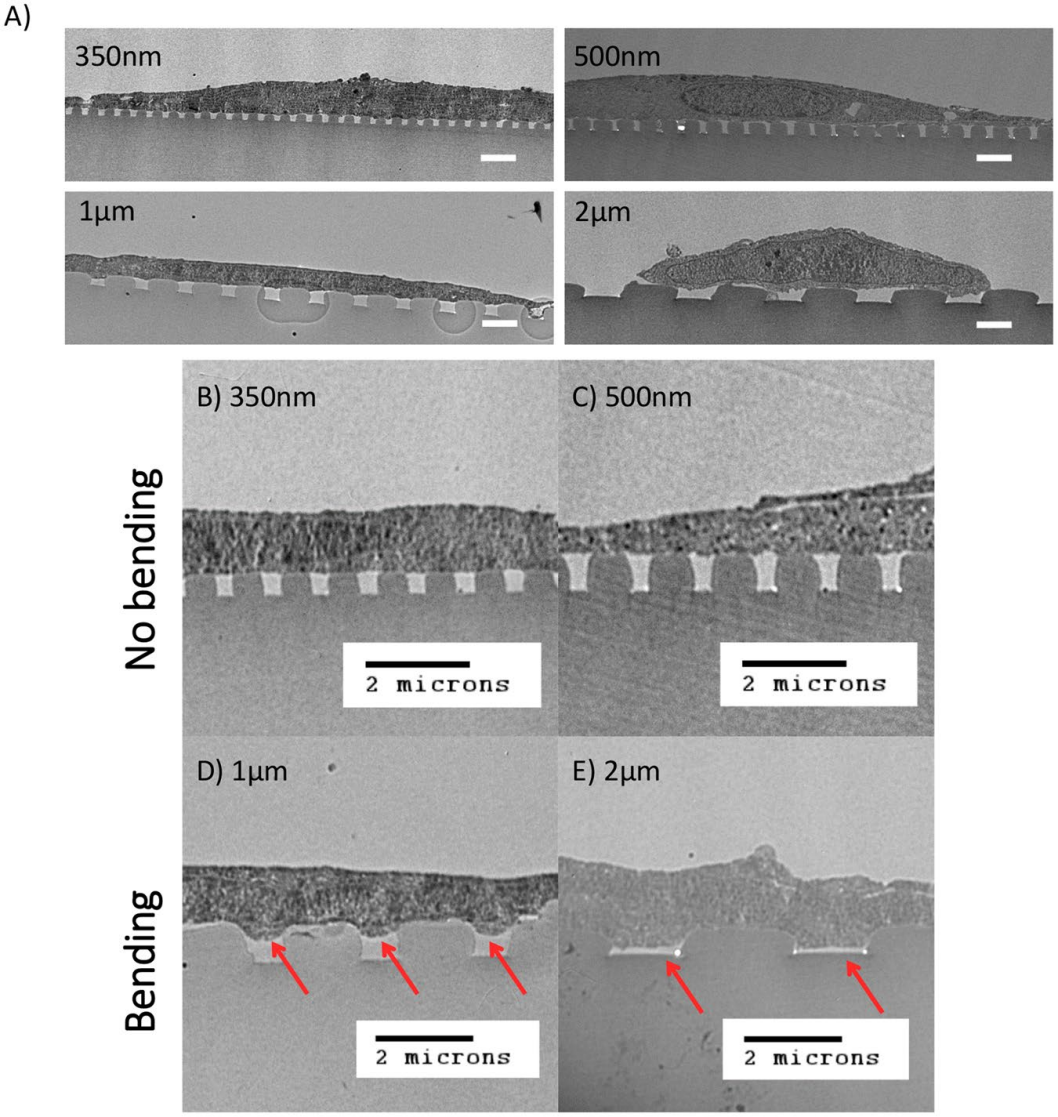
TEM images showing cross section profile of hMSC basal membrane bending on grating substrate. (**A**) Whole cell TEM images and (**B**–**D**) images zooming in on the cell membrane-substrate grating interface, where gap width of the gratings are as follows: (**B**) 350 nm, (**C***)* 500 nm, (**D**) 1 µm and (**E**) 2 µm. For cells on wider gratings, the basal membrane not only bent deeper into the grooves (red arrow denotes membrane bending into grooves) but also at high frequencies. (Scale bar, 2 µm).

Human mesenchymal stem cells (hMSCs) were seeded and cultured on gratings of different gap widths from 350 nm to 2 μm. At the end of 7 days, the samples were fixed and stained for viewing by transmission electron microscopy (TEM). Images of whole cells on gratings of different gap widths are shown in Fig. 4A. After zooming in on TEM images showing the cross-sectional profile (Fig. 4B–E), we observed that the hMSC had adhered onto the substrate with the cell basal membrane invaded into the gaps between the gratings (Fig. 4, red arrows). While this membrane-bending phenomenon was observed for all gratings widths, it was more extensive on the wider gratings. On 350 nm and 500 nm gratings, membrane bending was slight or non-existent (Fig. 4B,C). On the other hand, the membrane bending on the wider 1 μm and 2 μm gratings was more extensive and in some instances the cell filled up the entire groove space (Fig. 4D,E).

Even though the above observations suggested that membrane bending is modulated by grating width, it was also possible that the curvature of the ridges may play a role as membrane bending was not entirely absent over all grooves of the smaller 350 nm and 500 nm gratings. It had appeared that there was just a higher tendency for the membrane to bend as grating width increased. We proceeded to determine if there exist a threshold value for grating width above which membrane bending would always occur as well as the relationship between membrane bending, gap width and ridge curvature.

### Mathematical model for membrane bending

**Figure 5:**
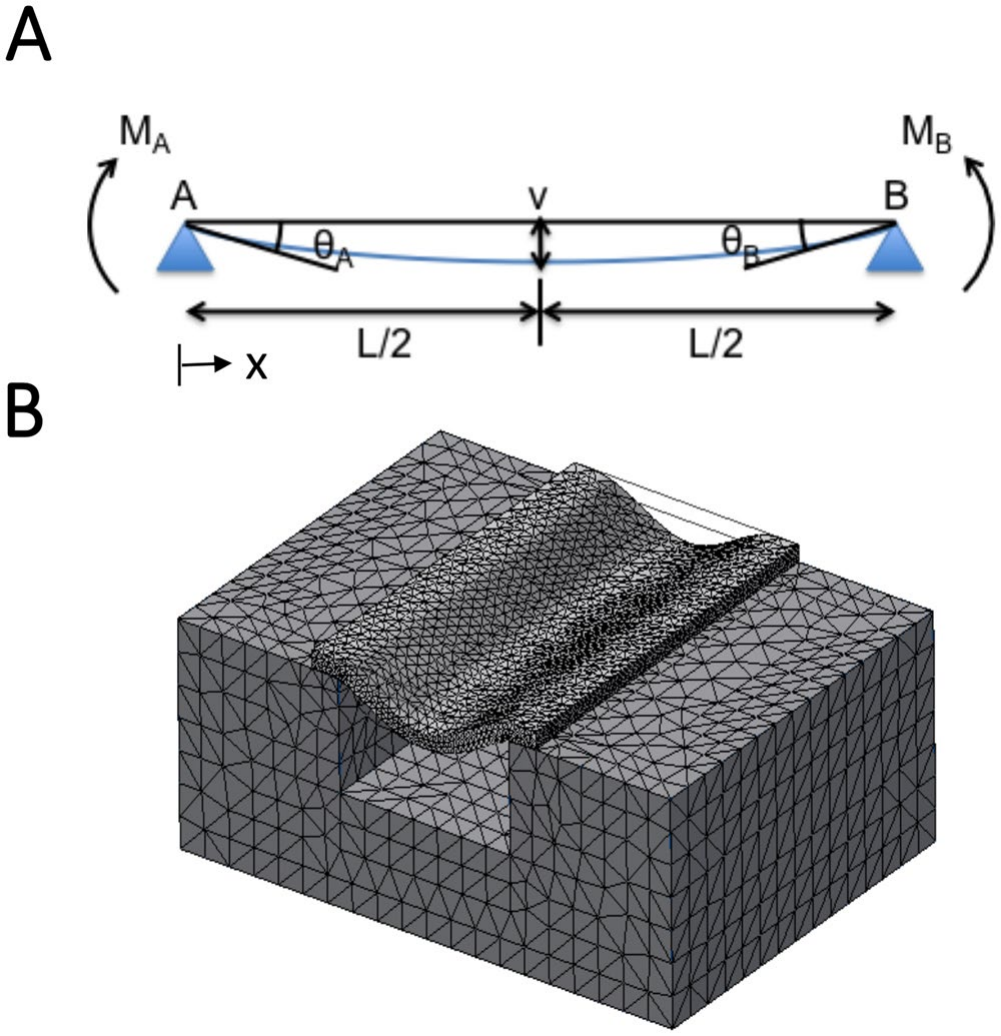
Theoretical and finite element models for membrane bending. (**A**) Membrane bending model modified from beam bending theory. A section of the membrane spanning the grating groove of length *L* is represented as a linear elastic beam with both ends (points *A* and *B*) freely supported by the adjacent grating ridges. The beam bends (blue line) under a uniformly distributed load *q* and the tangents to the membrane at point *A* and *B* make angles *θ*_*A*_ and *θ*_*B*_ respectively with horizontal line *AB*. The vertical displacement of any point *x* along the beam is denoted by *v* and is maximum when *x* = *L*/2 and *θ*_*A*_ = *θ*_*B*_. (**B**) Three dimensional finite element model simulation that was used to validate the results from the theoretical model for beam bending.

In order to understand this membrane bending phenomenon and the effects of grating groove width and membrane-bending angle, we developed a membrane-bending model, which was modified from the standard beam bending theory. In this model, a section of the membrane spanning the grating groove of length *L* is represented as a linear elastic beam with both ends (*A* and *B*) freely supported by the adjacent grating ridges (Fig. 5A). As indicated by the blue line, it bends into the grating groove space under a uniformly distributed load *q* that represents the driving force within the cell which pushes against the membrane. The extent of membrane bending is a result of the balance of forces due to this driving force and the bending rigidity of the membrane. If the tangents to the membrane at point *A* and *B* make angles *θ*_*A*_ and *θ*_*B*_ respectively with horizontal line *AB* (initial position of the membrane with no deflection), the vertical displacement of any point *x* (along the beam with reference to point *A*) from this line is given by

1 $$v=-\,\frac{q{x}^{2}}{24EI}({L}^{2}-2Lx+{x}^{2})-\frac{{\theta }_{A}}{{L}^{2}}({L}^{2}x-2L{x}^{2}+{x}^{3})-\frac{{\theta }_{B}}{{L}^{2}}(L{x}^{2}-{x}^{3})$$

where *q* is the intensity of a uniformly distributed load and *EI* is the flexural rigidity of the beam. In this case, the flexural rigidity of a beam, defined as the force couple required to bend a non-rigid structure to a unit curvature, is an indication of its resistance to bending and analogous to the bending modulus of the membrane. In our calculation, we have neglected surface tension effects as they are expected to have a small contribution compared to deformations due to bending given the geometries we are considering. When *θ*_*A*_ = *θ*_*B*_ = *θ*_*M*_, the maximum deflection of the beam occurs at *x* = *L*/2, where the normalised maximum deflection is given by

2 $$\frac{d}{L}=\frac{\max \,v}{L}=\frac{q}{24EI}(\frac{{L}^{3}}{16})+\frac{{\theta }_{M}}{4}$$

Details of the derivation of the above model and Eq. 2 can be found in the Supporting Material.

In order to validate the mathematical model derived for membrane bending in Eq. 2, we used a three-dimensional finite element model to simulate membrane bending (Fig. 5B). A membrane of known dimensions was supported at the two ends by fixed supports, and a known uniformly distributed force *q* was applied to the top surface of the membrane. The resulting deflection *d*/*L* was obtained and compared to the deflection value obtained from Eq. 2. Although the constructed model is one-dimensional, the results of the three-dimensional finite element bending model agree with the derived mathematical model for membrane bending (results not shown), suggesting that though actual membrane bending in cells occurs in two-dimensional space, our one-dimensional model is sufficient to characterize the observed bending phenomenon.

### Membrane deflection is dependent on membrane bending angle and grating gap width and validation of membrane-bending model against experimental data

The phenomenon of membrane bending into grating grooves was further analysed by quantifying the profile of the membrane that spanned over individual grating grooves. In each case, we measured the maximum membrane deflection (*d*), grating groove width (*L*), membrane bending angle (*θ*_*M*_) and substrate bending angle (*θ*_*S*_).

**Figure 6:**
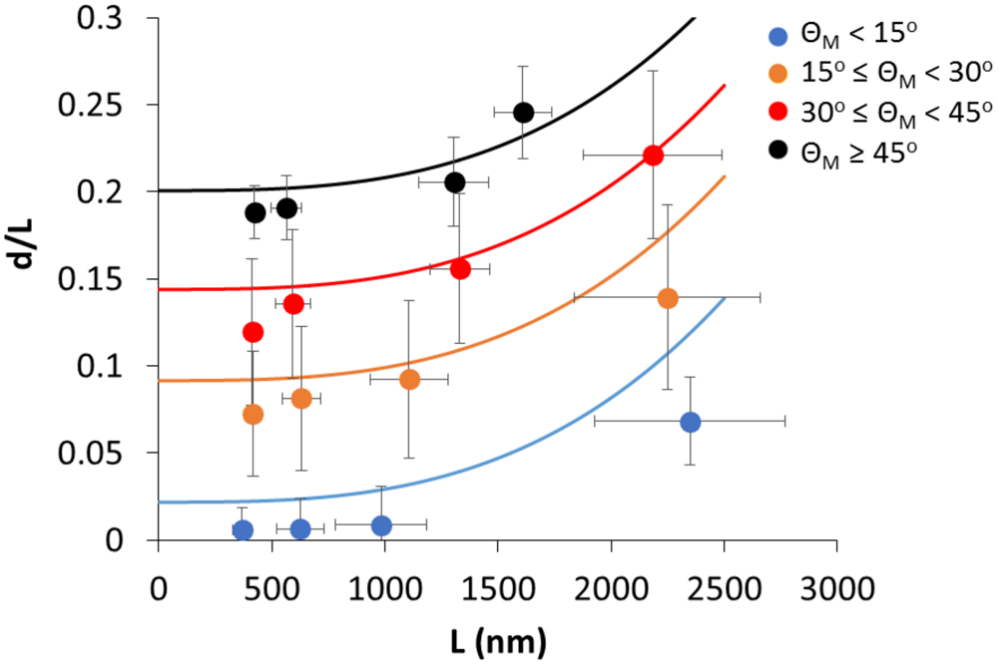
Plot of mean normalised maximum deflection ratio (*d/L*) versus mean grating groove width (*L*) for experimental data (dot plots) from gratings with widths of approximately 350 nm, 500 nm, 1 µm, and 2 µm segregated by membrane bending angle (*θ*_*M*_) into the following four groups: (i) *θ*_*M*_ < 15°, (ii) 15 ≤ *θ*_*M*_ < 30°, (iii) 30° ≤ *θ*_*M*_ < 45° and (iv) *θ*_*M*_ ≤ 45°. Theoretical prediction plots based on Eq. 2 are denoted by solid lines (Bars denote standard deviation, N ≥ 14 cells, with at least 8 membrane-ridge pairs in each cell).

Based on data collected from gratings with widths of 350 nm, 500 nm, 1 µm, and 2 µm, a graph of normalised maximum deflection ratio (*d/L*) versus grating groove width (*L*) was plotted as shown in Fig. 6. The experimental data was segmented based on *θ*_*M*_ into four groups (*θ*_*M*_ < 15°, 15° ≤ *θ*_*M*_ < 30°, 30° ≤ *θ*_*M*_ < 45° and *θ*_*M*_ ≤ 45°) to investigate the effect that *θ*_*M*_ has on *d/L*. In this case, although either *d* or *d/L* could be used as a matter of convenience, the latter was chosen as a non-dimensionalised quantity. The resulting plot showed that there appeared to be some clustering effects suggesting higher membrane deflections *d/L* in groups with larger membrane bending angles *θ*_*M*_. Within each of the four groups of data segregated by membrane bending angles *θ*_*M*_, there was also an apparent trend towards higher membrane deflection *d/L* on wider gratings *L*. Hence, membrane deflection is dependent on both membrane bending angle and the grating groove width.

To explain how membrane deflection is dependent on both membrane bending angle and the grating groove width in the observed experimental data, four theoretical graphs based on the mathematical model for membrane bending are plotted (Fig. 6, solid lines). The membrane bending model is validated by comparing the predicted maximum normalised deflection *d/L* in Eq. 2 with the experimental data as shown in Fig. 6. Corresponding to the observed clusters, the data was divided into the four subsets with the corresponding mean values of *θ*_*M*_ from the experimental datasets used in the model, which are 5°, 21°, 33° and 46° respectively. In the calculation of the predicted *d/L*, the values of load intensity *q* and flexural rigidity *EI* were estimated from known mechanical properties of living cells. The source of membrane bending could be due to several factors, such as intracellular pressure^30^ and actin polymerization forces pushing against the cell membrane^31^. As such, the value of *q* was calculated to be 0.03 N/m, assuming a uniform distribution of 300 Pa of intracellular cytosolic pressure^32,33^ over an elongated cell 100 μm in length. On the other hand, the value of *EI* can be obtained by considering the energy required in bending the membrane into unit curvature, which is 1.4 × 10^−15^ J in this case for a lipid bilayer with a bending modulus of 7 × 10^−18^ J^34^. Assuming this was the strain energy stored in a deflected beam under uniform load as given by *U* = *q*^2^*L*^5^/240*EI*, where *L* = 2 μm is the beam length, the value of *EI* was calculated to be 9.5 × 10^−21^ Nm^2^. These values for *q* and *EI* were substituted into the membrane bending model in Eq. 2 and plotted in Fig. 6.

As shown in Fig. 6, *d/L* predicted by the model exhibited a similar trend as the mean *d/L* calculated from the experimental data in all the four different ranges of *θ*_*M*_ - the value of *d/L* increases with a larger membrane bending angle *θ*_*M*_. As shown in Eq. 2, the normalised maximum deflection *d/L* is directly proportional to both *L*^3^ and *θ*_*M*_, which suggested that the extent of membrane deflection is dependent on both groove width *L* and membrane-bending angle *θ*_*M*_.

### Membrane bending angle is dependent on substrate bending angle

**Figure 7:**
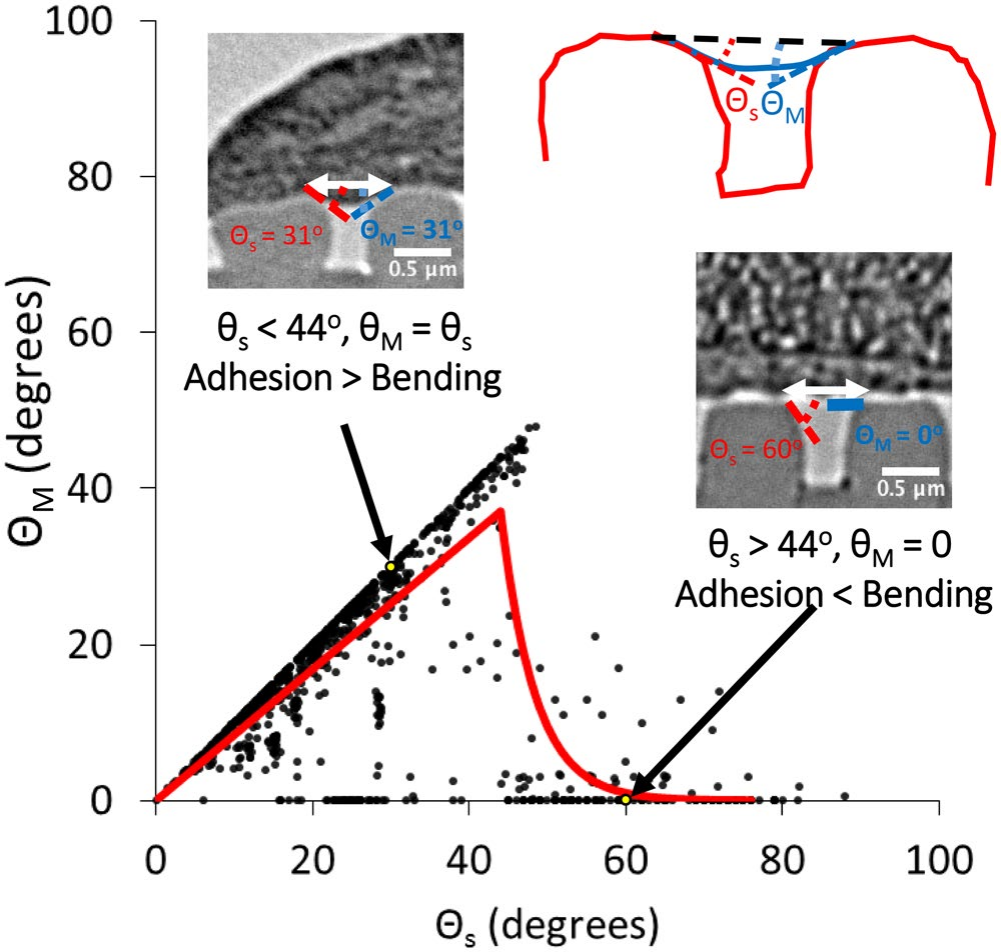
Plot of membrane bending angle (*θ*_*M*_) versus substrate bending angle (*θ*_*S*_). Black dots refer to experimental data points, while yellow dots refer to experimental data points as illustrated by the TEM inset images. Red line denotes the best fit linear and exponential decay lines to the two experimental data regimes for *θ*_*s*_ ranging from 0 to 44° and above 44° separately.

As the hMSC membrane is supported by the gratings at both ends, we further probed the possibility that the curvature at the top edge of the gratings might affect the extent of membrane bending by plotting the membrane bending angle (*θ*_*M*_) against the substrate bending angle (*θ*_*S*_) as shown in Fig. 7. To characterize the data, we obtained the best fit for the data to an initially linear regime and a subsequent exponential decay regime according to the following equations:

3 $${\theta }_{M}=a{\theta }_{s},for\,{\theta }_{s}\le b$$

4 $${\theta }_{M}={ab}^{\ast }\,e[\frac{-({\theta }_{s}-b)}{c}],for\,{\theta }_{s}\ge b$$

where *a*, *b* and *c* are constants and are equal to 0.8433, 44° and 4.397° respectively. These values are obtained from minimizing the squared errors of prediction for the curves denoted by Eqs 3 and 4 to the experimental data in Fig. 7.

As shown in Fig. 7, there exist a threshold value at *θ*_*s*_ ≈ 44°, at which the dependency of *θ*_*M*_ on *θ*_*s*_ changed. For *θ*_*s*_ < 44°, a clear trend showed that *θ*_*M*_ increased almost linearly with *θ*_*s*_. This observation can be explained by a balance of two forces – the adhesion force between the membrane and substrate, as well as the restoring force due to membrane bending energy. In the regime where *θ*_*s*_ is less than 44 degrees, membrane bending occurs as the membrane is close enough to the substrate such that the adhesion force is able to overcome the restoring force. In the other regime where *θ*_*s*_ is more than 44 degrees, the substrate is not in close proximity to the membrane to provide sufficient adhesion force to overcome the restoring force. As a result, the membrane remains unchanged in its initial non-deflected state. Hence this correlation again supports the notion that the membrane bending angle is dependent on substrate bending angle.

### Membrane bending model predicts membrane deflection regime

**Figure 8:**
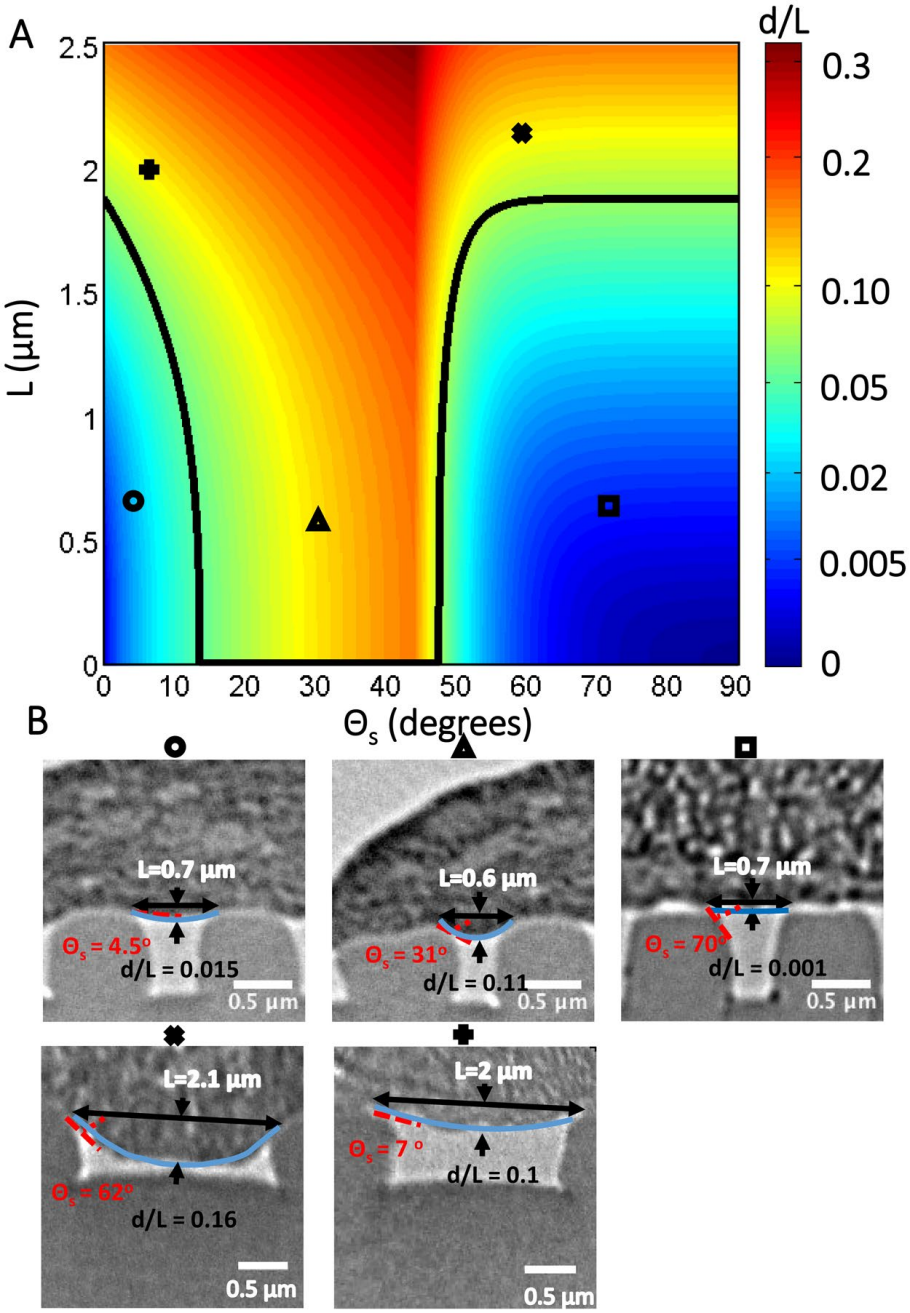
Plot of grating gap width (*L*) versus substrate bending angle (*θ*_*S*_) showing the extent of membrane deflection (*d/L*) into the gratings. (**A**) Colormap showing the varying degree of membrane deflection *d/L* with respect to *L* and *θ*_*S*_, with *black line* denoting the regime where *d/L* = 0.05. (**B**) The TEM images for experimental cases with *L* and *θ*_*S*_ values matching the corresponding symbols o, +, x, Δ and ϒ found in *(A)*.

By incorporating the relation between *θ*_*M*_ and *θ*_*s*_ determined previously into the membrane bending model from Eq. 2, we computed the regime of grating groove width and substrate bending angle in which membrane deflection occurs. This was done by first using the fitted experimental data obtained in Fig. 6 to obtain a value for *q*/24*EI* of 1.2 × 10^17^ m^−3^. By using this value of *q*/24*EI* obtained from the experimental data and substituting it into Eq. 2, the phase diagram in Fig. 8 of the extent of membrane deflection *d/L* as a function of grating gap width (*L*) versus substrate bending angle (*θ*_*S*_) is obtained.

In the phase diagram shown in Fig. 8, the model predicts that, for a given groove width of approximately more than 1.9 μm, membrane bending will occur regardless of the substrate bending angles. Below this threshold value, where the gap width is less than 1.9 μm, any bending of the membrane will depend on the substrate bending angles. At low substrate bending angles, the membrane is sufficiently supported by the substrate to avoid bending. As the slope at the top edge of the gratings increases above 13 degrees, the adhesion force between the membrane and the substrate causes the membrane to deflect downwards and bending to occur. In the last case, where the substrate bending angle is greater than 44 degrees, the membrane bending energy becomes high enough to overcome the attraction between the membrane and substrate, and the membrane is restored to its original non-bent configuration. As a result, there is a step change in the value of the threshold back to 1.9 μm for this region. This threshold value of approximately 2 μm in the size of gratings for membrane bending is an indication that the overall bending rigidity may be influenced by the cross-linked actin filaments in the cortical cytoskeleton, given that the persistence length of the actin filament is in the order of 10 μm^35,36^. Hence this model highlights the influence of gap width and curvature and predicts the conditions required for membrane deflection.

## Discussion

In this study of membrane bending on gratings, we have adopted the novel approach of direct viewing of sample cross section profile using TEM. While it was straightforward to carry out the sample preparation (cell fixing, dehydration and resin embedding), the major technical challenge lies in the selection of a suitable material for the substrate. During the sectioning process to cut the hardened resin block into thin slices, if the substrate material stiffness is not matched to the embedding resin, the slices will tear and separate at the interface. After trials with different materials, we have succeeded in overcoming this problem by using Spurr’s resin for the substrate as well as for embedding. As the patterning of Spurr’s resin involved using soft PDMS mold and additional mechanical force had not been applied to assist polymer filling to the PDMS mold, gratings with curved and un-sharp edges were observed in the AFM characterization. Nonetheless, the feature of curvature enable the investigation of the edge curvature in membrane deflection. Using Spurr’s resin as the substrate, we were also aware the potential artifacts during the dehydration and resin embedding processes. Patterned Spurr’s resin samples with and without cell-seeded were used to test and develop the fixature, dehydration and embedding protocol. To minimize the potential artifacts, we also substituted propylene oxide with ethanol in the standard preparation protocol as propylene oxide dissolves the resin substrate. Propylene oxide is used in standard preparation to displace the dehydrating agent and facilitate infiltration of the resin.

As the gratings are made from Spurr’s resin which have an elastic modulus in the order of Gigapascals^27,28^, they are assumed to be rigid and not deformable by the cell membrane or cytoskeleton forces. The substrate bending angle for each ridge is not caused by the driving force, but is an inherent curvature of each ridge due to the fabrication process. The hMSC basal membrane was shown to bend into the grooves of gratings with different widths. Based on data obtained from quantification of the membrane bending profile, this phenomenon was found to depend on both the groove width and the membrane-bending angle. By considering the membrane as a simple beam, a model based on Eq. 2 was then developed to explain this phenomenon of membrane bending. This model only includes the mechanism for membrane bending, without ridge deformation nor substrate bending, which is assumed to be negligible due to the rigidity of the substrate. However, we have shown experimentally (Fig. 7) that membrane bending angle is dependent on substrate bending angle, perhaps due to balance of the adhesion force between the membrane and substrate as well as the restoring force of the membrane due to membrane bending. The model was verified to be accurate in modeling the trends of increasing membrane deflection with larger membrane bending angle. This model can also predict if the basal membrane will bend into the grating groove for any given grating width and ridge curvature. In this case, membrane bending is predicted to occur on all gratings larger than 1.9 µm as well as on gratings smaller than 1.9 µm provided that the ridge curvature is between 13 and 44 degrees.

The above membrane-bending model was derived from the standard beam bending theory, which assumed isotropic linear elastic beam material and small beam deflection. It did not account for the non-linear elasticity in biological membrane and the relatively large deflection observed in the current dataset. In its current form as a simplified linear system, the model was validated when it predicted *d/L* that mirrored the trends observed in the experimental data and provided insights into the factors that influenced the process of membrane bending on gratings, which could include intracellular pressure or actin polymerization protrusive forces.

In order to improve the accuracy of the model in matching the experimental data, it could be revised to include the effects from other higher order dependencies. In addition, given that the experimental work was carried out over a period of 7 days, the cell membrane can be considered sufficiently stiff to justify the use of this model as the viscoelastic timescale of the membrane is much shorter, in the order of tens of microseconds^37^. Even if the whole cell is taken into consideration, its viscoelastic timescale is only in the range of one to tens of seconds^38^, depending on the cell type and cytoskeletal structure^39^, as well as the rate of application of mechanical load^40^.

Although we have shown that hMSC basal membranes bend into grooves depending on the size of width in between grooves and groove curvature, how membrane bending affects downstream cellular responses in hMSCs, such as cell proliferation or differentiation, requires a transduction of the physical topographical signal into an intracellular one. This could occur through curvature sensitive proteins such as the BAR-domain proteins, some of which upregulate the Rho family of GTPases^41^. Membrane bending could in turn activate topography sensor proteins, such as the BAR-domain proteins^42^ or stretch-sensitive channel proteins. The BAR-domain proteins are involved in the sensing of the curvature of substrates as well as in cell membrane dynamics^43^. It is known that some BAR-domain proteins result in the activation of proteins in the Rho family of GTPases^41,43^, which couples membrane bending brought about by substrate topography to intracellular signalling. This could in turn initiate a downstream signalling cascade to bring about further downstream cellular responses.

One of the critical members of the Rho family of GTPases is Rho, which is important in actin cytoskeleton regulation. Upregulation of Rho results in increased levels of phosphorylated myosin light chain^44^, promoting acto-myosin stress fiber formation in the cell. These stress fibers bind to focal adhesion complexes located on the basal membrane of the cell^45^. The change in stress fiber formation potentially provides a mechanism by which membrane bending brought about by substrate topography could be relayed as an intracellular mechanical signal.

For substrate topography to effect gene regulation, the physical topographical signal needs to first be transduced into an intracellular signal, which eventually activate genes within the nucleus. Cytoskeletal structures such as actin stress fibers bind to various nuclear proteins, such as the Sun and Kash proteins^35,46^, providing a direct mechanical link between the cell basal membrane and the nuclear membrane. Shivashankar *et al*. have shown nuclear shape and size changes can be brought about through the stresses in stress fibers in the cytoplasm^47^, which could in turn result in changes in gene expression^48^. Similarly, we have observed the presence of nuclear membrane bending in the direction of grating grooves in TEM images where cell basal membranes bend into grating grooves as well. In images where cell membrane bending is absent, nuclear bending in the direction of the grooves is not seen. This suggests that membrane bending brought about by substrate topography potentially brings about nuclear shape changes through the direct cytoskeletal mechanical link between the cell basal membrane and the nuclear membrane^49^. This change in nuclear shape could then elicit upregulation of genes responsible for various cellular processes.

It is known that culturing hMSCs on topographical grooves results in increased cell body elongation and alignment along the axis of the grooves as well as increased extent of neuronal differentiation in a previous study of hMSCs on groove gratings with various widths^17^. Moreover, the extent of elongation, alignment increases with decreasing groove width. When cultured on gratings with 350 nm and 1 µm widths (which are below 1.9 µm), hMSCs often do not exhibit basal membrane bending into the grooves, while the extent of cell body alignment and elongation is high. However, when hMSCs are grown on groove gratings with widths of 10 µm (which are above 1.9 µm), basal membrane bending into the grooves almost always occurs, while the extent of cell body elongation and alignment is low. These results suggest that membrane bending of hMSCs into the grating grooves might possibly reduce the extent cell body alignment and elongation in the axis of the grooves. The hMSC cell body alignment and elongation on grating grooves have also been linked to neuronal differentiation^17^.

A future study could include the investigation of how physical properties of topographical groove gratings direct downstream cellular processes such as cell proliferation through the presence of cell basal membrane bending into the grooves. For example, the mechanism of how the presence of hMSC cell basal membrane bending into topographical groove gratings potentially reduces the extent cell body elongation and alignment along the groove axis could be studied. The bending of the basal membrane could be transduced into an intracellular signal, specifically the promotion of acto-myosin stress fiber formation as demonstrated in a previous study of the neuronal differentiation of human embryonic stem cells on gratings^50^, which mechanically links the cell and nuclear membrane, bringing about changes in nuclear shape. The changes in nuclear shape could then bring about the up or downregulation of genes responsible for various cellular processes such as cell proliferation or differentiation. Through the construction of our membrane bending model which predicts the cell membrane deflection regime on grating grooves with different widths and curvature, in future studies we can potentially predict if a grating groove constructed with a particular width and curvature can bring about these downstream cellular processes based on cell membrane bending into the grooves.

### Data availability

The raw data used to generate the figures and tables in this manuscript are available from the corresponding or the first author upon request.

## Electronic supplementary material

Supplementary Information
