# Supplementary material for: Human mesenchymal stem cell basal membrane bending on gratings is dependent on both grating width and curvature
Source: Sci Rep. 2018 Apr 24;8:6444. doi: 10.1038/s41598-018-24123-6 (PMC5915387; doi:10.1038/s41598-018-24123-6)

**Supporting Material**

Title

Human mesenchymal stem cell basal membrane bending on gratings is dependent on both grating width and curvature

Author names

Yukai Zeng ^1^, Sum Thai Wong ^2,3^, Soo Kng Teo ^3^, Kam W. Leong ^4^, Keng-Hwee Chiam ^1,*^ & Evelyn K.F. Yim ^2,5,6,*^

Affiliations

^1^ Bioinformatics Institute, A*STAR, Singapore 138671, Singapore

^2^ Department of Biomedical Engineering, National University of Singapore, Singapore 117583, Singapore

^3^ Institute of High Performance Computing, A*STAR, Singapore 138632, Singapore

^4^ Department of Biomedical Engineering, Columbia University, New York, NY 10027, USA

^5^ Mechanobiology Institute, National University of Singapore, Singapore 117411, Singapore

^6^ Department of Chemical Engineering, University of Waterloo, Waterloo, ON, N2L 3G1, Canada

*Correspondence: [chiamkh@bii.a-star.edu.sg] and [eyim@uwaterloo.ca]

***Derivation of membrane bending model***

***x***

***L***

***q***

**FIGURE S1**: Standard beam of length *L* with uniform distributed load *q*.

In Fig. S1, the differential equation of the deflection curve of a standard beam is given by

$\frac{d^{2}v}{dx^{2}}=\frac{M}{EI}$ (1)

where *v* is the deflection at any point *x* along the beam, *M* is the bending moment and *EI* the flexural rigidity (*E*: Young’s modulus, *I*: Moment of inertia).

For a beam of length *L* with uniform load of intensity *q* acting throughout the span of the beam, the bending moment at a distance *x* is given by

$M=\frac{qLx}{2}-\frac{qx^{2}}{2}$ (2)

By substituting Equation (2) into Equation (1), we obtain

$ELv''=\frac{qLx}{2}-\frac{qx^{2}}{2}$ (3)

Solve for v by integrating twice with respect to x and considering boundary conditions $v'\left(\frac{L}{2}\right)=0$ and $v\left(0\right)=0$ in the evaluation of the constants of integration, we get

$v=-\frac{qx}{24EI}\left(L^{3}-2Lx^{2}+x^{3}\right)$ (4)

For beam bending with additional curvature boundary conditions specified, we modified the standard beam bending model by adding moments *M_A_* and *M_B_* to account for the given angles of rotation *θ_A_* and *θ_B_* at both ends of the beam as shown by Fig. S2.

***A***

***L/2***

***v***

**FIGURE S2**: Beam bending with uniform distributed load and curvature boundary condition. The beam supported at both ends (*A*, *B*) with length *L* and maximum deflection *v*. Moments *M_A_* and *M_B_* act at point *A* and *B* respectively to produce deflection angle *θ_A_* and *θ_B_*.

***L/2***

***B***

***θ_A_***

***θ_B_***

***M_A_***

***M_B_***

Using the principle of superposition, the overall deflection observed in this modified model can be deemed to be the sum total of all deflections produced by these individual loads acting separately: a uniform distributed load, two equal and opposite moments *M_B_* at both ends, and a net clockwise moment *M_o_ = M_A_ – M_B_* at point *A*.

The overall deflection *v* is therefore given by

(5)

Substitute *M_o_ = M_A_ – M_B_* into Equation (5) and simplify to get

(6)

Differentiate with respect to *x*

(7)

Setting the boundary conditions and we obtain

(8)

and

(9)

Substitute Equations (8) and (9) into Equation (6) and simplify

(10)

When , $\theta_{M}$being the membrane bending angle, where maximum normalized deflection is

$\frac{d}{L}=\frac{\max v}{L}=\frac{q}{24EI}\left(\frac{L^{3}}{16}\right)+\frac{\theta_{M}}{4}$ (11)

***Basal membrane bending angle (θ_M_) and substrate angle (θ_s_) characterization***

In the determination of the bending angles from from the TEM images, the first critical step in the measurement is forming a straight line between the points where the cell membrane comes into contact with the top surface of adjacent grating ridges. In all images, the background pixel intensity is much lower than the grating and cell pixel intensities (Fig. S3A), so the two points where the cell membrane detaches from the grating ridges have the same intensities as the background and can be determined at the pixel level of accuracy (Fig. S3B, *red dots*). A line is then connected to points *A* and *B*, denoting the distance *L (*Fig. S3C, *black line*).

**FIGURE S3**: *(A)* TEM images of hMSC on Spurr’s resin substrate, and *(B)* determining point of contacts *A* and *B* between basal membrane and substrate and *(C)* straight line distance *L*.


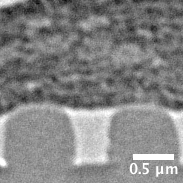


**A**

**B**


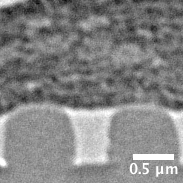


***A***

***B***

**C**


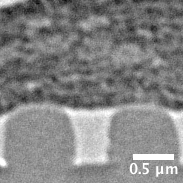


***A***

***B***

***L***

The next critical step is identifying the surface of the membrane and gratings so that a tangent to each surface at the points *A* and *B* where the cell membrane detaches from the grating ridges can be fitted and the bending angles subsequently determined. In this step, the thresholding method used to identify the membrane and grating edges affects the measured membrane and substrate bending angles. To give confidence in the accuracy of the thresholding of the images, we used three different thresholding methods in ImageJ (NIH, Bethesda, MD, USA). They are the Huang, IsoData and Otsu thresholding methods. The average difference in angle measurements by these three methods are around the 1-2 % range (Fig. S4). After thresholding, ***θ_M_*** is obtained as the angle between the line tangent to the curve of best fit at the membrane edges at the points of contact, and the line *AB*. ***θ_s_*** is obtained as the angle between the line tangent to the curve of best fit at the substrate edges at the points of contact, and the line *AB*.

**FIGURE S4**: Effect of image segmentation method (Huang, Otsu and IsoData) on the obtained substrate and membrane bending angles θ*_S_* and *θ_M_*.


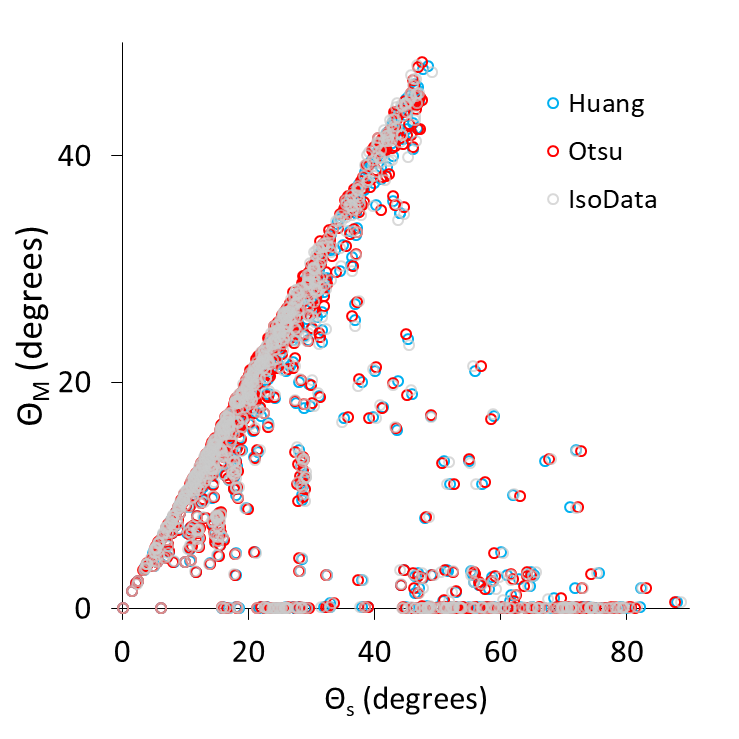


***Integrity of hMSCs and substrate grating samples after sectioning***

To ensure the integrity of the grating samples remain intact, we ensure that the fixation of specimen preserves the sample as close to the native status as possible. The sample were washed in warm serum free medium before fixing to avoid introducing shock to cells from changes in temperature or chemical environment. In the resin infiltration step, the pre-polymer infiltrate throughout the samples and subsequently cured and crosslinked to hardened the sample to provide mechanical support during sectioning and subsequent handling.

Although deformation during sample preparation process may be possible, such defects will be detected by the damages in the intracellular organelles, cell membrane and cytoskeletons. Therefore, the integrity of the intracellular structures were confirmed to be intact (Fig. S5) before accepting the samples for the subsequent TEM measurement.

**FIGURE S5**: Observing and ensuring the integrity of intracellular organelles in a hMSC after sectioning before accepting the samples for the subsequent TEM measurement.


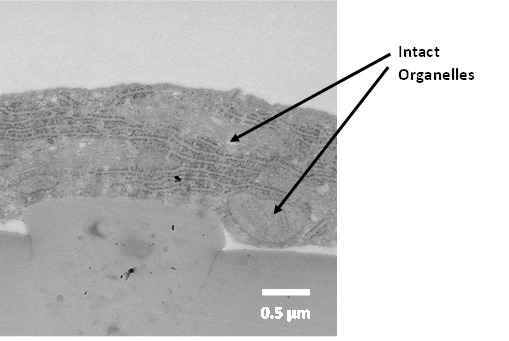

Supplement: Supplementary file 1 — Supplementary Information [file 41598_2018_24123_MOESM1_ESM.docx]
